# Supporting healthcare in rural communities in Thailand: An exploratory qualitative study to understand the role and current mental health practices of village health volunteers

**DOI:** 10.1371/journal.pone.0320559

**Published:** 2025-03-27

**Authors:** Chonmanan Khanthavudh, Annmarie Grealish, Vasiliki Tzouvara, Mary Leamy

**Affiliations:** 1 Florence Nightingale Faculty of Nursing, Midwifery & Palliative Care, King’s College London, London, United Kingdom; 2 Faculty of Medicine Ramathibodi Hospital, Ramathibodi School of Nursing, Mahidol University, Bangkok, Thailand; 3 Department of Nursing and Midwifery, Health Research Institute, University of Limerick, Limerick, Ireland; Mahidol University Faculty of Tropical Medicine, THAILAND

## Abstract

**Introduction:**

Village health volunteers (VHVs) are the backbone of primary healthcare in many low-and-middle-income countries, including Thailand, where healthcare professionals are scarce. Previous studies looking at their role have been broader and lacked a specific mental health focus. In 2019, Thailand introduced a policy endorsing a recovery orientation in mental health care, however, the potential for VHVs to implement the approach remains underexplored. This study aims to: [1] describe VHVs’ mental health practices, [2] explore stakeholders’ perspectives on these practices, and [3] understand stakeholders’ views on their potential to deliver recovery-oriented community care.

**Method:**

This exploratory qualitative study involved nineteen semi-structured interviews conducted between August 2023 and March 2024 in a rural subdistrict of Northern Thailand. Participants included ten VHVs, four nurses, four caregivers, and one individual with mental health conditions. Purposeful and snowball sampling techniques were used. Reflexive thematic analysis was used to analyse interview data. Official documents related to VHVs’ job descriptions, training, and recruitment policies were also examined to understand the scope of the role.

**Results:**

The analysis identified three main themes: [1] Mental health practices and roles perceptions, highlighting variability among VHVs; [2] Organisational constraints on mental health practice in the community, demonstrating limited policy support and training for VHVs; and [3] Factors influencing the implementation of recovery-oriented approaches by VHVs, including barriers such as stigma and workload, and enabling factors such as specialist training and professional support.

**Conclusions:**

This study reveals that VHVs in Thai rural communities prioritise physical health due to policy adopting a biomedical approach and limited training on providing mental health care. A range of culturally adapted approaches are needed to expand and enhance the contribution that VHVs can make to improving the quality of life of individuals experiencing mental health conditions in rural communities in Thailand.

## Background

Global mental ill-health, especially severe and enduring mental illnesses, causes poor quality of life, early mortality, and substantial economic consequences [1,2]. The World Health Organisation (WHO) has strongly advocated for community-based mental health care to increase accessibility, reduce stigma, support community reintegration, improve quality of life, and protect the human rights of individuals from coercive treatments in institutional care [3,4]. However, a pressing global shortage of mental health professionals, particularly in low- and middle-income countries (LMICs), poses a substantial barrier to delivering effective mental health services [5]. Thailand, an upper-middle-income country in Southeast Asia, is challenged by a scarcity of trained personnel, despite integrating mental health services into its broader healthcare system [6,7]. Although Thai mental health policy has long favoured community-based care [6,8,9], a national mental health survey study revealed that only 11.5% of individuals with mental health issues and substance use disorders had access to healthcare services, with only 3.7% accessing support from healthcare professionals [10].

Community health workers have emerged as vital assets in primary healthcare, advancing both national and global health agendas. This has included achieving universal health coverage as outlined in the Sustainable Development Goals in LMICs, such as Thailand, Indonesia, India, Brazil, and Ghana [11,12]. A promising solution to staff shortages lies in enhancing the capacity of community workers to deliver basic care in collaboration with stakeholders [7,13]. Such workers are known internationally by different terms, including village health workers and lay health workers. In Thailand, they are officially designated as Village Health Volunteers (VHVs) according to national policy. Originating in the 1970s, these non-professional volunteers have been integral to primary healthcare delivery in Thailand’s communities [14,15], and there are currently over one million VHVs serving communities across Thailand.

Residents who wish to become VHVs are nominated by community residents. There is no formal education level required for selection. They receive thirty-seven hours of foundational training in core topics and six hours of training in elective topics. Training covers core areas such as subdistrict health management, infectious disease surveillance and control, health promotion, mental health, and maternal health alongside special topics like diabetes mellitus and disability [14]. Each volunteer is given responsibility for caring for 10–20 households [16]. They are required to work at least one day per week or at least four days per month, are reimbursed for their time, and receive basic oversight and assistance from primary healthcare professionals [17]. Once qualified, VHVs perform a range of duties including preventing dengue fever, monitoring pregnant women, and supporting people with chronic diseases such as diabetes and hypertension through blood pressure and blood sugar monitoring. They also provide older adult care to increase activity, decrease loneliness and prevent conditions such as Alzheimer’s disease [18]. Evidence suggests that globally, community health workers and lay providers can improve care for underserved areas and lessen disparities in mental health [19]. VHVs also have the potential to increase the accessibility and acceptance of mental health care, while helping to address the shortage of mental health personnel [7,20,21].

WHO has outlined the importance of good quality person-centred, rights-based, recovery-oriented mental health services in the community [22]. Recovery-oriented approaches involve supporting people to (re)gain meaning and a sense of identity, empower self-directed living, foster hope for the future and heal from trauma [23]. Despite being a relatively new concept in Asia, recovery-oriented care was endorsed in Thai national mental health policy (2019–2020) and prioritised in the Department of Mental Health’s 5-year operational plan (2023–2027) as one of five flagship issues. The approach is operationalised through strategies such as reducing stigma, promoting mental health literacy, enhancing vocational rehabilitation, and developing peer support programmes. Training programmes, such as Job Coach initiatives, and the integration of recovery principles into service standards further support implementation [24,25]. However, specific evidence on stakeholder perspectives, including user groups, advocates, healthcare professionals, and VHVs regarding this approach was not found. Cultural variations in personal and family values, beliefs, and lifestyles are likely to affect the acceptance, development, and implementation of recovery-oriented care in non-western countries like Thailand [26–28]. Moreover, stigma continues to be a significant barrier to mental health care in the country, influencing public attitudes and help-seeking behaviours [29]. Cultural factors, such as beliefs in karma or spirits causing mental illness, and societal stereotypes that often portray individuals with mental health conditions as dangerous, contribute to this issue [30,31].

To ascertain the possibility for extending and enhancing the VHV role in mental health, it is important to improve our understanding of how these roles currently function in resource-constrained communities. Several previous studies have explored the importance of Thai VHV roles in specific health emergencies such as the avian influenza outbreak [32], and the COVID-19 pandemic [33–35]. However, to date, little research has explored the practice of VHVs in mental health, the views of those who support them, or indeed on the experiences of individuals living with mental health conditions who receive their care. Given the shortage of mental health personnel in Thailand, particularly in remote rural regions [36], the potential implications of engaging the VHVs to deliver recovery-oriented approaches also need to be explored.

### Aim

This study has three overarching aims:

[1] To describe VHV’s roles and responsibilities in providing mental health care to individuals with mental health conditions in rural Thailand through an exploration of stakeholders’ views, including VHVs, carers/individuals with mental ill-health, and healthcare professionals.
[2] To explore the experiences and perspectives of stakeholders regarding VHVs’ mental health practices in caring for individuals with mental health conditions.
[3] To understand stakeholders’ views on the potential for VHVs to extend their role and deliver recovery-oriented approaches in the community.

## Materials and methods

### Patient and public involvement (PPI)

Involving patients and the public enhances the representation of public perspectives, making the study more relevant to their needs and improving its overall quality [37]. By leveraging local knowledge, public and patient involvement fosters a sense of ownership and strengthens research planning and implementation, particularly in unfamiliar settings, creating meaningful impacts on both the process and the individuals involved [38]. In this study, two PPI advisors, who were VHVs, collaborated on the research process. They were asked to provide feedback on the interview questions, appropriate language to use with local participants, and guidance on the recruitment process.

### Design

An exploratory descriptive qualitative design was chosen to gain an in-depth insight into the current mental health roles of VHVs and the potential to extend their role in recovery-oriented approaches [39]. Data triangulation was achieved by interviewing VHVs, carers and/or individuals with mental health conditions, and healthcare professionals to elicit multiple perspectives. The study also used methodological triangulation, employing both interview data from VHVs and stakeholders, alongside an analysis of documentation on job descriptions, recruitment policies and training of VHVs in Thailand. Such an approach allows the cross-validation of interpretations and conclusions, ensuring the robustness and credibility of the study while reducing the potential for biases [40]. Reporting followed the consolidated criteria for reporting qualitative research (COREQ) checklist [41].

### Study setting and selection of participants

This study was conducted in a rural district of Lampang province, Northern Thailand. Lampang province’s VHVs gained national recognition for their effective COVID-19 vaccination efforts, collaborating closely with healthcare professionals and community networks [42]. However, little is known about their role in other areas, particularly in mental health care, where mental health professionals are limited. This study targeted up to nineteen participants, a sample size considered adequate based on Malterud et al.’s [43] concept of ‘information power’. This concept suggests that when the research question is highly relevant to participants’ experiences, fewer participants are needed. Given the study’s specific focus on mental health in resource-constrained rural communities with a limited number of healthcare professionals and the depth of the interviews, this sample size was deemed sufficient to provide meaningful insight. Interview participants were identified using purposive and snowball sampling [44]. Purposeful sampling was used to ensure that participants with specific knowledge, experience, or perspectives related to the study’s objectives were selected [45]. This approach is aligned with the study’s objective of exploring the roles and experiences of those directly involved in mental health care in rural communities. Snowball sampling was additionally used to increase the number of participants [44]. VHVs, individuals with mental health conditions, and their carers were recruited from eleven rural villages in Lampang province. The healthcare professionals were recruited from a subdistrict health promoting hospital (SHPH) and a mental health unit located within a district hospital in the province. The eligibility criteria for each type of participant are outlined in Table 1.

**Table 1:** Eligibility criteria for four different types of participants.

Types of participants	Inclusion criteria	Exclusion criteria
**VHVs**	Being a registered VHV Having worked with at least one household of individuals with mental health conditions Aged 18 years old or above Sufficient ability to speak, read and write Thai and able to provide informed consent Residing in one of 11 villages in a subdistrict	N/A
**Individuals with mental health conditions**	Identified by the VHVs/Healthcare professionals from the SHPH and district hospital as having a history of mental health conditions and being sufficiently well at the time of interview Aged 18 years old or above Sufficient ability to speak, read and write Thai and able to provide informed consent Residing and receiving care from VHVs in one of 11 villages in a subdistrict	Identified by the VHVs/Healthcare professionals from the SHPH and district hospital as having suicidal risk or risk behaviours Having comorbidities with substance use disorders
**Carers**	Family member, close friend, or carer who has direct experience in caring for individuals with mental health conditions living in one of 11 villages in a subdistrict Aged 18 years old or above Sufficient ability to speak, read and write Thai and able to provide informed consent	N/A
**Healthcare professionals**	Healthcare professionals who work at the SHPH or a mental health unit in a district hospital Sufficient ability to speak, read and write Thai and able to provide informed consent	N/A

N/A =  Not applicable, SHPH =  Subdistrict Health Promoting Hospital, VHV =  Village Health Volunteer.

### Recruitment

Recruitment was carried out from July 2023 to March 2024. Flyers containing the research objectives, participant criteria, and the contact details of the first author (CK) were posted at the SHPH. The study area has a limited number of healthcare professionals, with only three working at the SHPH and two at the mental health unit in the district hospital. Given this constraint, we were able to recruit three general nurses and one mental health nurse from these facilities who agreed to participate. To further facilitate the recruitment and access of a diverse range of VHVs, the director at the SHPH was approached to provide potential candidates. The director provided a list of twelve VHVs with experience of working with individuals with mental health conditions. These individuals were directly contacted by the researcher through telephone to ascertain their interest in the study. Of these twelve, two did not respond to our calls, and one was subsequently deemed ineligible due to limited contact with individuals with mental health conditions. A PPI member provided the name of another VHV who met the eligibility criteria, which increased the sample size to ten.

The recruited mental health nurse provided a list of eligible individuals with mental health conditions and their carers, including those caring for both eligible and ineligible individuals. Five eligible individuals with mental health conditions were approached in total, but one declined to participate with no specific reason provided, and another relocated out of the area. Of the three remaining individuals, two were very quiet, timid, and unsure how to answer, preferring their caregivers to answer for them. Engaging individuals with mental health conditions was challenging, particularly in rural areas. This difficulty may stem from cultural factors such as shame, embarrassment, or stigma around discussing mental health, as well as the inaccessibility. Consequently, only one individual with mental health conditions spoke directly to the researcher. Thus, the final interviews included four carers: two who spoke on behalf of the individuals they cared for, one carer of the individual who participated directly, and one carer whose care recipient was ineligible for the study. All participants were provided with the Participant Information Sheet and their contact details were shared with the first author (CK) to organise a convenient time to explain the study and address any questions. Verbal consent was recorded at the beginning of each interview.

### Data collection

#### Interviews.

Semi-structured interviews were conducted between August 2023 and March 2024. The interview guides (see S1 Appendix) were adapted slightly depending on the stakeholder group. Core questions included: (1) the extent of current VHV activity in mental health care, (2) views on the knowledge and training of VHVs in relation to mental health, and (3) perspectives on utilising VHVs to deliver recovery-oriented approaches. The first two core questions were developed with careful consideration of the research objectives and the stakeholders’ roles. The third question was informed by a review that highlights the growing integration of recovery-oriented practices in mental health care across Asia [26]. The interview guides were initially developed in English and then translated into Thai and further reviewed with a bilingual doctoral student (RC) for clarity, applicability, and relevance, ensuring the use of neutral and respectful language to avoid stigmatising terms. The guides were piloted with academic and clinical colleagues in London and Thailand, and two PPI members. Subsequently, minor adjustments to the flow and order of the questions were made.

Interviews were primarily conducted by telephone, with face-to-face and virtual options also made available to participants. This approach was chosen based on participant preferences, which likely included geographical challenges and COVID-19-related physical distancing measures in Thailand. Azad et al. (2021) examined the experiences of individuals with common mental disorders or multimorbidity through mobile phone interviews and identified that this method provides benefits such as increased flexibility, anonymity, a more balanced power dynamic, and enhanced self-disclosure and emotional openness [46]. These benefits made telephone interviews a practical choice for our study, providing a private and comfortable setting that helped reduce barriers or anxiety often associated with face-to-face interactions. Open-ended questions and a flexible interview guide with probing techniques were used to encourage participants to elaborate on their responses and share detailed insights. Interviews were conducted in Thai by the first author (CK), who is a female native Thai speaker. Each interview was audio recorded with permission. It was transcribed verbatim in Thai and then translated into English by CK, with close discussion with a bilingual doctoral student (RC) to address potential mistranslations and ensure that the nuances of the language differences were accurately conveyed. The interview duration ranged from 38 to 60 minutes, with an average interview time of 47 minutes (totalling 14 hours 49 minutes).

#### Documents.

VHV documents were identified through searches on government health department websites. This included official Thai government publications, regulations and reports on VHVs’ job description, recruitment policies, and mental health training modules.

### Data analysis

Data were extracted from the documents by the first author (CK) into a table, which was checked by the bilingual doctoral student (RC). Reflexive thematic analysis [47,48] was employed in this study to identify shared meanings, enabling comprehension of a group of experiences, thoughts, or behaviours across the dataset [49]. This is a flexible data-driven method that was chosen as it is appropriate for exploring patterns in narratives and it is iterative rather than linear in fashion, as the researcher can repeatedly revisit raw data and critique codes and themes over a period of time [48]. NVivo software (version 14) was employed to organise all transcribed interviews and documentary data.

Braun and Clarke’s [47,48] six-step framework was used to identify and generate themes, which involved data familiarisation, generating initial codes, generating initial themes, reviewing themes, defining and naming themes, and producing the report. Commencing with the data familiarisation, the first author (CK) transcribed the recordings, read the transcripts, and listened to the recordings repeatedly to gain a deep understanding of the data’s content. Subsequently, codes were created line by line through a manual analysis of all interview transcripts by the author (CK), alongside simultaneous data collection with regular discussions among the research team (ML, AG, VT). Following the completion of all data collection, the author (CK) separately analysed data from each stakeholder group and the extracted document data to compare and contrast the findings. Basic codes were generated by the author (CK) to highlight similarities and differences within the data, and codes with similar underlying meanings were then combined. These codes were merged into themes and subthemes through analytic memo writing, which were subsequently reviewed and modified for accuracy and coherence within the research context. Regular meetings with the research team were held to reflect and discuss the initial themes and subthemes. The themes were further refined, named and defined to precisely represent the dataset. Any disagreements during the analysis were resolved through discussion among the research team prior to the final theme generation. Lastly, the results were organised into a comprehensive summary of findings with supporting quotes, ensuring that the research questions were addressed. Throughout the analytic process, both Thai and English transcripts were utilised, acknowledging the nuances and subtleties inherent in language. Incorporating both versions enabled researchers to comprehensively capture and interpret these nuances, which enriched data analysis.

### Methodological rigor

This study adopted rigor by using the four criteria proposed by Lincoln and Guba [50]: credibility, dependability, confirmability, and transferability, which researchers typically agree constitute high-quality qualitative analysis (see Table 2).

**Table 2:** Trustworthiness of the qualitative data as recommended by Lincoln and Guba (1985).

Criteria	Researcher actions
Credibility	Data were triangulated using various data sources such as interview transcripts, document analysis, reflexive notes, and debriefing.
Dependability	A detailed record was maintained throughout the data collection process.
Confirmability	Codes, themes and subthemes were discussed among the researchers to enhance the depth and richness of interpretation and refine the final themes and subthemes.
Transferability	Purposive sampling was utilised, and a detailed description of the research setting and participant characteristics was provided.

Reflexivity: To enhance researcher reflexivity, the first author (CK) engaged in ongoing critical self-reflection, consistently recording reflections following each interview and throughout the data collection and analysis process [48]. This practice allowed the researcher to examine how their professional and personal background might shape their engagement with the data and the analytic process. The first author is a Thai PhD student, mental health nurse, and has a particular interest in mental health recovery within the community. A team approach was used to engage deeply with the data and refine analytic insights. Regular meetings were held to discuss coding the data set and the subsequent interpretation of this data. The research team (ML, AG, VT) was composed of female academic health professionals and experienced qualitative researchers in mental health. The first author (CK) had no prior experience working with VHVs in Thailand.

As a native Thai speaker with an understanding of Thai cultural norms, the first author adapted their approach to align with local practices, facilitating rapport-building with participants. In Thai culture, individuals are often addressed using terms that reflect their age, social standing, or professional role, such as “Aunty,” “Uncle,” “Sis,” or “Bro” for those older than the speaker. Participants were also addressed as “Doctor” even when they were nurses, a colloquial practice in Thailand that honours their expertise, particularly in rural areas. These culturally ingrained practices of respect and familiarity helped participants feel valued and comfortable sharing their experiences.

### Inclusivity in global research

Additional information regarding the ethical, cultural, and scientific considerations specific to inclusivity in global research is included in the Supporting Information (S2 Checklist).

### Ethical considerations

Ethical approval was obtained from the King’s College London Research Ethics Committee (Reference: HR/DP-22/23-36445), and data collection permission was also obtained from the Lampang Provincial Health Office (Reference: LP0033/4323). A minor amendment to expand the sample size was required and approved by the relevant ethics committees. The study complied with the Helsinki Guidelines to maintain standard research ethics [51].

## Results

### Participant characteristics

Nineteen participants were interviewed, and their demographic characteristics are presented in Table 3. All participants were of Thai ethnicity and identified as Buddhist. The VHVs were all female and were aged between 39 and 75 years. Experience as a VHV ranged between one and 23 years. One VHV served as both the VHV head and specialised in mental health. All four carers were female, aged between 50 and 70 years, and were mothers, wives, sisters or sisters-in-law of a person living with mental health conditions. The individual with mental health conditions was diagnosed 11 years prior, based on ICD-10 criteria, as reported by the mental health nurse.

**Table 3:** Participant demographic information.

Type of study participants	Village Health Volunteers	Healthcare professionals	Carers/Individual with mental health conditions
**Number of Interview participants**	10	4	5
**Sex**			
Female	10	3	4
Male	–	1	1
**Mean age (years)**	57	51	60
**Educational level**			
Primary school	6	–	2
Junior high school	1	–	1
Senior high school	2	–	–
Vocational school	1	–	1
Bachelor’s degree	–	3	1
Master’s degree	–	1	–
**Position**			
VHV’s Chairman	1	N/A	N/A
VHV’s Head	1	N/A	N/A
VHV’s Member	8	N/A	N/A
Registered nurse	N/A	3	N/A
Mental health nurse	N/A	1	N/A
**Year of experience**			
Average years of volunteering experience	12	N/A	N/A
Average years of working experience in the current workplace	N/A	17	N/A
Average years of being a carer	N/A	N/A	9

N/A =  Not applicable, VHV =  Village Health Volunteer.

Seven official documents were identified and analysed, as listed in the Table 4.

**Table 4:** Village health volunteer documents used for analysis.

No.	Document title	Selected data	Source
**1**	Regulations regarding village health volunteers of 2011	Job description, Person specification	Notification of Ministry of Public Health published in the Government Gazette
**2**	Criteria and procedures for selecting outstanding village health volunteers, 2019	Person specification	Notification of Ministry of Public Health
**3**	Entitlement to receive remuneration for village health volunteer services in community health activities for the fiscal year 2021	Benefit	Notification of Ministry of Public Health published in the Government Gazette
**4**	Operational standards for village health volunteers and reporting performance according to form (VHV.1) 2023	Job description/Requirement	Notification of Central Village Health Volunteer Promotion and Support Committee, Ministry of Public Health
**5**	Form VHV. 1	Monthly Performance Report	Ministry of Public Health
**6**	Role of village health volunteers specialised in community mental health, 2018	Training material	Department of Mental Health, Ministry of Public Health
**7**	Course on accessing services and caring for patients with psychosis for village health volunteers (Revised edition 2018)	Training material	Department of Mental Health, Ministry of Public Health

VHV =  Village Health Volunteer.

### Theme findings

Three main themes were identified from the analysis of interviews and informed by the analysis of documentation: (1) Mental health practices and roles perceptions of VHVs; (2) Organisational constraints on mental health practice in the community; (3) Factors influencing the implementation of recovery-oriented approaches by VHVs. Table 5 presents an overview of coding framework.

**Table 5:** Overview of coding framework.

Theme titles	Subthemes	Example of Illustrative codes
**1. Mental health practices and roles perceptions of VHVs**	Variability in mental health activities among VHVs	Mental health activities
		Medication is important
		Advanced mental health activities
		We’re not doctors
		Document 2, Table 4
	Perception of VHVs’ competence	Relationship between VHVs and others
		Positive attitude towards VHVs
		PMI/Carers’ negative attitude towards VHVs
**2. Organisational constraints on mental health practice in the community**	Limited policy support for mental health practice	Physical health over mental health
		Document 1, 3, 4, 5, 6, 7, Table 4
	Lack of mental health training provision	Frequency of training
		Not all VHVs have received mental health training
		Mental health is not a major problem
		Document 2, 6, Table 4
**3. Factors influencing the implementation of recovery-oriented approaches by VHVs**	Barriers	Stigma
		Low expectations towards PMI
		It’s not the role of VHVs
		Workload
		Differing personality and ability of VHVs
	Enabling factors	VHVs’ willingness to expand mental health work and cooperation
		Simplicity of new approaches
		Training needed
		PMI vary from person to person
		Organisational preparedness

HCP =  Healthcare professional, PMI =  People with mental ill-health, VHV =  Village Health Volunteer.

### Mental health practices and role perceptions of VHVs

This theme encompasses two subthemes which describe the VHVs’ current practice: (1) Variability in mental health activities among VHVs; (2) Perceptions of VHVs’ competence in mental health.

#### Variability in mental health activities among VHVs.

VHVs and nurses reported that the VHVs were broadly engaged in three types of mental health activities.

1. Biomedical activities: These activities were the most commonly undertaken and included monitoring medication adherence, screening for symptoms, observing any mental health deterioration, and identifying new cases. The importance of ensuring medication adherence in particular was emphasised by VHVs and healthcare professionals.

*“We need to ask them about the remaining quantity of medication, the number of days it covers, and when their next doctor’s appointment is.” (01VHV)*

2. Coordinator/management activities: Some participants spoke of the role VHVs played in coordination or management, such as being the first point of contact for individuals, conducting home visits, and coordinating with healthcare professionals.

*“I advise the relatives not to leave them alone (individuals with mental health conditions) and if the symptoms get worse, they should notify me immediately. I’ll coordinate with the hospital for treatment.” (02VHV)*

3. Therapeutic engagement/ supportive/ recovery-oriented activities: Some VHVs spoke about their involvement in activities such as promoting social inclusion (e.g., by encouraging participation in community activities), offering emotional support, providing active listening and engaging in conversations.

*“If I know they are stressed, I talk to them, comfort them.” (09VHV)*

However, the nature and extent of mental health work varied among VHVs, influenced by their individual experience and expertise.

Relevant documentation suggests that each village should have one VHV specialising in each of 12 care areas, including community mental health (Table 4, Document 2). These specialised VHVs receive priority for advanced training opportunities as a way of recognising their outstanding work and dedication. Reflecting this, one of the participants, who was an outstanding VHV in mental health, engaged in advanced tasks. This included the administration of medication, de-escalating disruptive behaviours, assisting with restraint, providing counselling, and organising appointments and community activities:

*“…I have to work on calming their emotions while waiting for Dr. XX (mental health nurse) and the police to arrive. If their emotions don’t settle, we administer medication to help the patient regain control.” (02VHV)*

In contrast, some general VHVs could only provide basic care, and acknowledged the need for specialised assistance from healthcare professionals:

*“We offer encouragement, we diligently care for them, consistently ask about their condition, how they take their medication. That’s about all we can do because we’re not doctors...” (07VHV)*

#### Perceptions of VHVs’ competence.

VHVs were viewed by participants as essential frontline workers in rural areas, with healthcare professionals providing guidance, support, and additional resources. All nurses emphasised the strong connections the VHVs have with both community residents and themselves, comparing these relationships to that of "siblings". Nurses also appreciated their dedication and described the mutual trust which contributed to a robust collaborative partnership between nurses and VHVs:

*“VHVs contribute a lot.... If someone they look after shows signs of not doing well, they have to refer them, or sometimes, if they’re unsure, they can consult with a nurse….We will quickly go to take care of it because VHVs are the first point that is close to the patients…. VHVs are indispensable. They are serving as vital components in caring for patients within the community, much like arms and legs.” (04Healthcare professional)*

Caregivers and individuals living with mental health conditions noted VHVs’ focus on improving physical health problems such as diabetes, hypertension, dengue fever, and COVID-19. However, they reported limited mental health support from VHVs. One carer explained:

“*VHVs don’t involve in taking care of things in this aspect. It’s just us taking care of everything ourselves...VHVs never took care of my brother… Since my brother first started showing symptoms, we’ve had to figure everything out on our own.” (02Carer/Individual with mental health conditions)*

For some, this led to limited trust in VHVs’ abilities to provide good mental health care, reinforcing their reliance on healthcare professionals.

*“…I don’t think they (VHVs) can take care of them (individuals with mental health conditions)…. The doctors should come from the mental health department as they are trained and knowledgeable about dealing with patients’ behaviour.” (05Carer/Individual with mental health conditions)*

### Organisational constraints on mental health practice in the community

This theme focuses on the major obstacles that prevent VHVs from reaching their potential in supporting individuals with mental health needs, namely (1) Limited policy support and (2) Lack of mental health training.

#### Limited policy support for mental health practice.

The main regulations governing VHVs outline only broad roles and responsibilities, such as disseminating health information, healthcare prevention activities, fostering community involvement, and coordinating with local organisations (Table 4, Document 1). Explanations of the specific mental health responsibilities for VHVs are limited to certain training materials (Table 4, Document 6 and 7). Some of these materials comprise information about monitoring mental health symptoms, screening for stress and depression, looking for developmental issues and referring to professionals for severe problems. A handbook for VHVs to enable them to provide support for psychosis was also identified. This stressed the importance of reporting “abnormal behaviour”, assessing risk factors for relapse, ensuring medication was taken and reporting to healthcare professionals. Additionally, the monthly performance form, which is submitted by VHVs to receive the government’s remuneration of 1000 baht (£22/$28), include activities such as motivating and encouraging patients to take care of their health, but does not specifically mention mental health care (Table 4, Document 3, 4 and 5).

Responses from participants also suggested that the practical involvement of VHVs in mental health care was limited. Some nurses in this study were clear that chronic disease was generally considered a greater priority than mental health in Thailand. This approach was therefore reflected in the types of mental health tasks that they allocated to VHVs:

*“Mainly, it’s chronic non-communicable diseases that result in a relatively high number of disabilities or deaths. We focus more on this aspect rather than mental health issues, which are not considered a top priority… Therefore, VHVs help identify who might have mental health conditions. However, we don’t go into much detail or depth regarding this.” (03Healthcare professional)*

#### Lack of mental health training provision.

Most VHVs reported not receiving specific mental health training. Although there were monthly meetings held, VHVs stated that the focus of their education was predominantly on physical health conditions:

*“Mostly, the training has been about hypertension, diabetes, elderly care, and general health issues. Mental health has not been a part of my training.” (07VHV)*

Some nurses reported that areas of basic mental health were covered in the mandatory training that were delivered to VHVs. Documents defining the roles of VHVs in mental health also provided information about the “3Ls” approach to screening for psychiatric problems in the community (Table 4, Documents 2 and 6). A nurse explained how they taught VHVs this basic approach to notice people struggling with mental health issues:

*“L1 is Look, observe and identify. L2 is Listen, pay attention and listen. L3 is Link, coordinate and refer. …. I’m not sure whether they can remember it or not, but I teach it every time.” (04Healthcare professional)*

Refresher training for existing VHVs on mental health appeared limited. Some nurses also acknowledged their own lack of confidence with providing such care. More advanced mental health techniques and training on suicide risk was reserved for VHV leaders and hospital staff, and the frequency appeared to vary, depending on specific village needs:

*“Some years, we have it (training), and some years we don’t. It depends on the occurrence of suicide events in that particular year. If it’s not a prevalent issue, we might not conduct training.” (03Healthcare professional)*

### Factors influencing the implementation of recovery-oriented approaches by VHVs

This theme captures the diverse factors to enhance the role of VHVs in mental health care through recovery-oriented approaches. Two subthemes were identified: (1) Barriers and (2) Enabling factors.

#### Barriers.

Stigma surrounding individuals with mental health conditions was identified as hindering VHV engagement in mental health care. One participant explained how, in local communities, mental health conditions are often referred to as a *“nerve condition” (06VHV).* A few VHVs and nurses expressed a fear of being harmed by individuals with mental health conditions, which was a hindrance to their engagement:

*“Skills in approaching and dealing with these patients are lacking. They (VHVs) are scared, you know. We are sometimes scared as well.” (01Healthcare professional)*

Counter to the ideas of hope and empowerment associated with recovery approaches, some VHVs held low expectations of individuals with mental health conditions. These individuals were perceived as withdrawn, incapable of working, or uninterested in participating in community activities, which underscored the potential risk of social exclusion for these residents.

Carers agreed that VHVs avoid caring for individuals with mental health conditions due to either a fear of being attacked or because of their belief that these individuals were incapable of recovering from their health condition:

*“I think initially they (VHVs) might have been afraid. Everyone saw Uncle’s mania, so they might have been scared and didn’t come…It’s like they viewed him as crazy, so they didn’t think he would get better.” (03Carer/Individual with mental health condition)*

Other VHVs and nurses displayed a poor understanding of the complexity of mental health, believing that people who appeared relatively *“normal” (01VHV)* or were isolated at home and engaging in unhealthy behaviours, but not causing trouble, did not need their help at all.

Another perceived barrier was VHVs’ lack of enthusiasm or capacity for taking on the role.

Some VHVs explained how their other responsibilities impacted their ability to fully engage in recovery-oriented approaches and contributed to an “*overwhelming workload” (07VHV)*.

*“My challenges include handling various aspects of work, not just mental health. I need to look after the elderly, those with disabilities, bedridden patients, and sometimes I have to perform wound care for patients at home, so they don’t have to go to the SHPH or hospital.” (02VHV)*

Some VHVs were unsure about how much they should even be involved in mental health. Others felt that this kind of care was *“outside their responsibilities” (05VHV)* and should be left to qualified mental health nurses, or that the burden should fall largely on the family and VHVs should simply guide family members:

*“Families, well, they’re the ones responsible for taking care. We are outsiders; it has to start from those closest to them.” (07VHV)*

Several nurses expressed concern about the capacity of some of the VHVs to extend their role to include recovery-type activities. They noted VHVs who were selected could be relatively elderly people who may not be as capable of understanding new approaches, undergoing specialised training or following more complex instructions:

*“…because in the district, there are many elderly VHVs. Communication and reading and writing abilities might be problematic for some. When we communicate or assign tasks for screening or assessment, even if we explain it 100%, they will only accept 50% due to their age.” (01Healthcare professional)*

#### Enabling factors.

Many VHVs expressed a positive attitude towards the development, introduction, and engagement of recovery-oriented care. They first emphasised the need for *“simplicity” (04VHV)* in the approaches and a comprehensive manual to facilitate ease of implementation. VHVs and nurses highlighted the importance of providing comprehensive mental health training to build VHVs’ competency and confidence. Mental health specialists were considered as appropriate to provide this training.

VHVs stressed the need for clear direction and support from healthcare professionals if they were to expand their role. Some suggested that it would be possible to give greater authority to VHVs by providing recognition for those who had undergone additional mental health training:

“*VHVs need acceptance…In my opinion, there should be a focus on certification for individuals with knowledge and understanding in the field of mental health…. The certification would serve as proof of their knowledge in the field…” (03Healthcare professional)*

Some VHVs reflected on the importance of respecting the distinct needs of each individual on their journey to recovery:

*“I think understanding each patient is important - because they’re all different.” (09VHV)*

Some carers explained how empathy, kindness, being persuasive, and having attentive listening skills were key attributes for those providing effective mental health care.

*“If VHVs treat them (individual with mental health conditions) with kindness, speak to them gently, there won’t be any issues at all” (02Carer/Individual with mental health conditions)*

Both nurses and VHVs suggested the need for further discussions within the subdistrict health promoting hospital. This would help to foster collective awareness in recovery approaches, achieve consensus on key priorities and organisational issues, and pilot approaches on selected VHVs before expanding the approach.

## Discussion

This study explored the roles of VHVs in delivering mental health care within rural communities of Northern Thailand and stakeholder attitudes to expanding their role into mental health using recovery-oriented approaches. The findings revealed that VHVs are primarily focused on biomedical tasks but also provide coordination and some therapeutic engagement and support. Notably, their mental health practices include screening and monitoring conditions, conducting home visits, providing emotional support, and coordinating care between local healthcare professionals and individuals with mental health conditions. This is similar to the role of lay community mental health workers in Indonesia [52,53]. However, while previous studies have highlighted positive service user experiences with lay community workers [52], our small sample of families in Thailand had little experience of receiving effective mental health support from VHVs. One participant, an experienced VHV, described engaging in advanced tasks such as administering medication and assisting with restraint, illustrating the complexity of the VHV role and potential ethical issues. This highlights the need for further training to ensure VHVs are adequately prepared to manage complex tasks, which is essential for maintaining competence, ethical standards, and trust in their roles.

Our analysis of documentation for the VHV role found limited policy or practice guidance on duties and responsibilities for mental health care. While mental health care is officially part of their role and supported by policy, it is often deprioritised in practice. Participants also reported that limited mental health tasks were assigned to them by professionals, which reflects the fact that physical health still takes precedence over mental health in community care in Thailand. Consequently, VHVs perceive mental health tasks as additional responsibilities rather than core elements of their role. This deprioritisation has a knock-on effect where VHVs have inadequate mental health training and therefore have poor knowledge, skills, and confidence. Indeed, this mirrors the situation in other LMICs, where limited knowledge, inadequate policy guidance, and organisational planning all hinder effective mental health service provision [54,55].

Our study suggests that there is still significant stigma surrounding mental health conditions, and VHVs and nurses have low expectations of individuals with mental health conditions. Such individuals can be perceived by workers as frightening, withdrawn, and incapable of work or recovery, reflecting a significant gap in mental health knowledge, as noted by WHO [22]. Our findings are also consistent with previous research conducted globally and regionally, including in China, Brazil, Kenya, India, and Indonesia, which shows that community health workers often lack adequate mental health knowledge and hold stigmatising attitudes towards those with mental health conditions [53,56–58]. For instance, in rural China, village providers similarly express concerns about patient violence and unpredictability [58]. Additionally, some VHVs and staff felt that their involvement with individuals with mental health conditions should be limited, and that families should be in charge of caring for their unwell relatives. This somewhat reflects the collectivistic values inherent in Asian cultures, where familial support and involvement are highly valued in caregiving roles [59]. However, relying solely on family support overlooks the potential of engaging with a multifaceted support system. Other findings also emphasised the complexity of evaluating mental health beyond apparent symptoms. For example, VHVs and carers in our sample did not always understand the dangers of individuals with poor mental health becoming socially isolated or engaging in harmful behaviours, such as smoking and excessive alcohol consumption. Clearly, stigma continues to hinder mental health care in Thailand, as evidenced by our study and supported by a broader review of stigma-related research across Asian societies and worldwide [22,28,29]. Programmes like SMART in India and RESHAPE in Nepal have successfully used social contact-based interventions to reduce stigma, though further research on their long-term effectiveness is needed [60]. Given these findings, urgent efforts are needed in Thailand to promote anti-stigma initiatives and foster wider understanding and acceptance of mental health issues and treatment [56].

Recent guidance from WHO [22] and the Lancet Commission on global mental health and sustainable development [61] calls for scaling up mental health services, particularly in LMICs. Similarly, Thai mental health policy supports a recovery-oriented approach, beginning with destigmatisation and promoting employment opportunities [25]. Although Thailand is still in the process of improving its mental health services, integrating mental health care into the existing primary healthcare system in line with WHO guidelines, as we are doing, has the potential to advance communities and society, increase accessibility, improve patient outcomes, reduce suffering, and promote dignity [4]. This could involve implementing pro-recovery programmes or training initiatives that provide continuous mental health education and skills development, along with ongoing supervision for VHVs [62]. Nurses and allied healthcare professionals in Thai primary care units play a pivotal role in supervising VHVs in the community. However, mental health presents a complex challenge even for these primary healthcare professionals, and it is paramount that there is ongoing training and support for the community-based workforce from mental health specialists.

VHVs highlighted the importance of specialised training provided by mental health professionals and of having clear instructions, guidance, and supervision. These concerns are consistent with findings from multi-site qualitative studies of non-specialist health workers delivering mental health care in LMICs such as India, Nepal, South Africa, Ethiopia, Uganda, and Ghana. It concluded that defined roles, trusted trainers, ongoing supportive supervision, and sufficient training were crucial for success [63,64]. These factors also enhance the potential of VHVs to bridge mental health service gaps in low-resource settings, as demonstrated by initial evidence in this study. VHVs play a crucial role in the mental health and recovery of individuals by offering emotional support and fostering social engagement. For example, one VHV shared that they encourage individuals to participate in community activities to help keep them engaged and prevent overthinking. This initial evidence highlights the significant impact of VHVs in promoting mental well-being and supporting recovery in their communities. However, interventions for individuals with mental health conditions or training programmes for VHVs to support these individuals must be culturally sensitive and context-specific. Indeed, adaptation is essential to ensure recovery-based approaches, which were originally developed in the West, meet the diverse needs of consumers from other parts of the world [28]. To tailor recovery-oriented practices to the Thai cultural context, strategies should consider Asian cultural values such as humility, respect for family, and religious beliefs, particularly Buddhism in Thailand. Methods like self-exploration and facilitative questioning should be adopted to accommodate varying literacy levels and ensure recovery concepts are understood within the cultural framework. These approaches align with Thai cultural norms and promote more effective recovery, as highlighted in the cultural adaptation of recovery-oriented practices in Asia [26]. Such initiatives should therefore be developed in partnership with VHVs, individuals with mental health conditions and their families, healthcare professionals, and local agencies. Evidence suggests the inclusion of stakeholders enhances the acceptability and local support for community-based mental health interventions in LMICs [13]. This participatory approach underscores the importance of community involvement in intervention development and ownership, which is crucial for long-term sustainability [13,65].

### Implications for future research, practice, and policy

This study highlights ways to strengthen the role of VHVs in mental health care in communities in Thailand and similar settings where lay people and VHVs play a critical role in supplementing primary care. This research underscores the necessity for practical initiatives to enhance VHV training, engage with communities to cultivate trust, dismantle stigma, and enhance mental health literacy. Several specific policy recommendations are necessary. First, policies should formalise the integration of mental health care into VHVs’ job descriptions, with clear guidelines and expectations for their mental health responsibilities. To further reduce stigma, interventions such as public awareness and social contact-based approaches should be promoted, and collaboration with local authorities is essential to strengthen community support for mental health care. Additionally, ongoing training and refresher courses must be mandated to keep the VHVs updated on mental health issues and recovery practices. To enhance the confidence and ability of VHVs in supporting mental health care, several simple changes are suggested. These include regularly discussing mental health during monthly meetings, incorporating more mental health topics into core training, and adding more mental health content to their monthly monitoring reports. These initiatives may help familiarise them with mental health care and encourage them to view it as an integrated part of their role. Moreover, it is crucial to enhance VHVs’ training programmes by developing specialised modules focused on mental health awareness, literacy and communication skills. Support systems, such as peer support for new VHVs, should also be implemented. Strengthening collaborations between the VHVs and mental health professionals through joint home visits and case discussions will improve service delivery. Future research to co-develop educational training programmes for VHVs and healthcare professionals on the benefits of recovery-oriented mental health care can equip them with the necessary knowledge and skills. Emphasising the core values of recovery, such as empowerment, hope, social integration, and self-determination, and contrasting these with traditional illness-focused approaches is essential. By shifting the focus from deficits to strengths, recovery-oriented approaches empower individuals to build on their abilities, promoting their rights, dignity, and self-worth. This approach fosters meaningful inclusion and active participation in society, helping to reduce stigma and create opportunities for individuals to contribute and lead fulfilling lives.

### Strengths and limitations

To our knowledge, this paper is the first qualitative study based in Thailand to investigate VHVs’ practices in community mental health and the potential for expanding their role to provide recovery-oriented approaches. However, our sample was dominated by female participants, and only one individual living with mental health conditions in our sample spoke directly to the researcher. While attempts were made to include as many individuals living with mental health conditions as possible, those we approached were either not interested in taking part or felt more comfortable letting their carer answer questions for them. It is important to focus on finding ways to empower individuals living with mental health conditions to share their views. Indeed, their experiences of mental health care by VHVs will be essential in the evaluation of future recovery-based interventions. Further studies should consider how best to collect data from these individuals. For example, interviewing people face to face in a place of their choosing and accompanied by trusted individuals could help to increase rapport. The small sample size and gender imbalance among participants may have influenced the predominantly negative perspectives from individual with mental health condition and carers. While the findings highlight certain patterns, a more diverse and larger sample could provide a more comprehensive picture. Additionally, the variation in VHVs’ roles and responsibilities reflects the diverse nature of their work and the differing needs of the communities they serve. VHVs were purposefully recruited from 11 villages, with the key criterion being that they supported at least one household with a member experiencing mental ill-health. Given the nature of VHV work, some VHVs may be more actively involved in mental health support, while others may primarily provide general health services.

Restricting participation to literate individuals, including carers, individuals with mental health conditions, and VHVs, may have excluded the perspectives of those with limited literacy, particularly in low-resource settings. Future studies should make greater efforts to ensure that all community members, regardless of literacy, have equal opportunities to participate. In addition, the health professionals recruited to this study were all nurses. While this is the professional group who have the most contact with VHVs, it would have still been useful to have gathered the attitudes of other professionals, such as medical staff, who work with them. Moreover, the participants who chose to take part in this study may have had different opinions on a VHV’s role in mental health care compared with those who did not take part. Despite meticulous translation efforts and the inclusion of the original Thai and English transcripts and documentation, some subtleties from the conversations may not have fully translated due to the complexity of conveying culturally specific words [66]. Finally, a further limitation of this study is the absence of involvement from individuals with mental health conditions in the PPI process as their engagement could have provided valuable insights.

## Conclusion

This qualitative study highlights limited and primarily biomedical-focused mental health practices among VHVs in rural Thai communities, with insufficient policy support. It is evident that physical health is prioritised over mental health in community care. This prioritisation, stigmatisation and limited mental health support compromise the treatment and quality of life for individuals with mental health conditions in the community. Our findings suggest the need to develop interventions which support VHVs’ mental health practices in Thai communities. It is critical to emphasise mental health awareness and literacy and person-centred training to promote recovery-oriented practices. Policymakers should formalise mental health tasks within their job descriptions, ensuring clear guidelines and expectations. Collaborations with healthcare providers, mental health specialists, local authorities, and community stakeholders to co-develop tailored training programmes are essential for improving service delivery and ensuring the long-term sustainability of community-based interventions. This study provides novel insights into enhancing the role of VHVs in rural Thai settings and offers valuable perspectives for similar contexts in LMICs. Future research should explore the VHVs’ mental health roles in other regions of Thailand and involve a broader sample of individuals experiencing mental health conditions to further inform policy and practice.

## Supporting information

S1 Appendix Interview guide for semi-structured interview. (DOCX)

S2 Checklist Inclusivity in global research. (DOCX)
