# Supplementary material for: Supporting healthcare in rural communities in Thailand: An exploratory qualitative study to understand the role and current mental health practices of village health volunteers
Source: PLoS One. 2025 Mar 27;20(3):e0320559. doi: 10.1371/journal.pone.0320559 (PMC11949336; doi:10.1371/journal.pone.0320559)
Supplement: S1 Appendix — (DOCX) [file pone.0320559.s001.docx]

**S1 Appendix.** Interview guide for semi-structured interview

**1. Village health volunteers (VHVs)**

- What is your role or responsibilities specifically in caring people with mental illness in the community? (PROBE: What services, care, support do you provide for members of the community?)
- What type of support do you provide and how often do you provide care or support for people with mental illness or their carers? (PROBE: Could you describe the activities related to mental health practice?)
- How do community residents with mental illness access mental health care?
- What kind of training have you received? (PROBE: Have you received any training specific to mental health? If yes, what training? who provided the training?)
- Do you feel that you have enough knowledge and skills to take care of community residents with mental illness?
- What could be done to improve mental health care in the community?
- Can you describe good practices and challenges within your role/responsibilities when providing care to people with mental illnesses? (PROBE: What aspects of your work are you most proud of? What are the issues related to your work?)
- How are you addressing the challenges you mentioned previously?
- What is your opinion on using VHVs to deliver recovery-oriented intervention to people with mental illness in the community?
- What do you see as the opportunities and challenges of implementing the intervention by VHVs here? (PROBE: What suggestions do you have for making it work?)
- How confident are you in your ability to perform this role? (PROBE: What do you think other VHVs would think about implementing the intervention by VHVs?
- What would you need to take on this role? (PROBE: what kind of training would you need to carry out this role effectively?)

**2. Carers**

- Can you tell me about your experiences in receiving care from VHVs in relation to caring for your relatives with mental illness? (PROBE: What kind of care did you receive from VHVs and how often?
- How do VHVs provide care to your relatives with mental illness? (PROBE: Could you describe the VHVs’ roles and activities related to mental health practice?)
- What are the issues that you are dealing with caring people with mental illness?
- How are you addressing these challenges?
- What kind of mental health service is currently available to you in the community? (PROBE: Do you feel that it is enough? Is there any further support that you would like?)
- Do you think VHVs have enough knowledge and skills to take care of community residents with mental illness?
- What kind of mental health service do you expect to receive from VHVs?
- What could be done to improve mental health care in the community?
- What is your opinion on using VHVs to deliver recovery-oriented intervention to people with mental illness in the community?
- What do you see as the opportunities and challenges of implementing the intervention by VHVs here? (PROBE: What suggestions do you have for making it work?)
- How confident are you that VHVs could carry out this role? (PROBE: What do you think other people would think about this? what kind of training would VHVs need to carry out this role effectively?)

**3. Individual with mental illness**

- Can you tell me about your experiences in receiving care from VHVs? (PROBE: What kind of care did you receive from VHVs and how often?)
- How do you seek mental health care in the community?
- Could you describe the VHVs’ roles and activities related to mental health practice?
- What kind of mental health service is currently available to you in the community? (PROBE: Do you feel that it is enough? Is there any further support that you would like?)
- Do you think VHVs have enough knowledge and skills to take care of community residents with mental illness?
- What kind of mental health service do you expect to receive from VHVs?
- What could be done to improve mental health care in the community?
- What is your opinion on using VHVs to deliver recovery-oriented intervention to people with mental illness in the community?
- What do you see as the opportunities and challenges of implementing the intervention by VHVs here? (PROBE: What suggestions do you have for making it work?)
- How confident are you that VHVs could carry out this role? (PROBE: What do you think other people would think about this? what kind of training would VHVs need to carry out this role effectively?)

**4. Healthcare professionals**

- In your opinion, what are the main VHVs’ roles or responsibilities specifically in caring people with mental illness in the community?
- How do community residents with mental illness access mental health care?
- What services, care, support do VHVs currently provide for members of the community? (PROBE: Could you describe the VHVs’ activities related to mental health practice?)
- What kind the interactions or roles you have with VHVs? (PROBE: Can you tell us some of those activities?)
- What type of support do you provide and how often do you provide support for VHVs?
- What kind of training have VHVs received? (PROBE: Have VHVs received any training specific to mental health? If yes, what training? who provided the training?)
- Do you think VHVs have enough knowledge and skills to take care of community residents with mental illness?
- What could be done to improve mental health care in the community?
- What is your opinion on using VHVs to deliver recovery-oriented intervention to people with mental illness in the community?
- What do you see as the opportunities and challenges of implementing the intervention by VHVs here? (PROBE: What suggestions do you have for making it work?)
- How confident are you that VHVs could carry out this role? (PROBE: What do you think other people would think about this? what kind of training would VHVs need to carry out this role effectively?)
- What role do you think you can support VHVs in providing care for people with mental illness in community?
